# Catastrophic Antiphospholipid Syndrome after Orthotopic Liver Transplant

**DOI:** 10.1155/2022/6209300

**Published:** 2022-05-05

**Authors:** R. Okeke, J. Lok, R. Wells, M. Wycoff, A. Engelhardt, J. Bettag, C. O'Leary, T. Hallcox, M. Nazzal

**Affiliations:** Saint Louis University, St. Louis MO 63108, USA

## Abstract

**Background:**

Catastrophic antiphospholipid syndrome (CAPS) is an autoimmune thrombogenic disorder of small and large vessels caused by autoantibodies against phospholipids and phospholipid-binding proteins. This severe form of antiphospholipid syndrome (APS) presents clinically with simultaneous life-threatening multiorgan thrombosis and the presence of two or more persistent antiphospholipid antibodies (APL) confirmed on testing 12 weeks apart. *Case Presentation*. We describe a case report of a 66-year-old woman with detected antinuclear antibodies (ANA) pretransplant diagnosed with CAPS following orthotopic liver transplant. The patient had acute respiratory failure; Doppler ultrasound and CT angiogram confirmed thrombosis in the hepatic artery, subsequent occlusion of the jump graft, and a splenic infarct. Hypercoagulability workup showed elevated levels of anticardiolipin IgG and beta-2-glycoprotein IgG/IgM and positive lupus anticoagulant, treated with steroids and anticoagulation. The patient was discharged after one month and was transitioned from heparin to life-long warfarin.

**Conclusion:**

Our patient provided a standard presentation of CAPS with abnormal pretransplant levels of antinuclear antibodies (ANA). Although there have been studies investigating the relationship between anticardiolipin antibodies and lupus anticoagulants and APS, the relationship between pretransplant positive ANA or antimitochondrial antibodies (AMA) and CAPS has yet to be explored. Further studies will be needed to determine the significance of these antibodies. We recommend preoperative APL testing for patients with positive ANA and AMA at preliver transplant presentation.

## 1. Introduction

Antiphospholipid syndrome (APS) is an autoimmune thrombogenic disorder affecting small and large vessels in which phospholipid-dependent clotting assays, negatively charged phospholipids, and phospholipid binding proteins are attacked by autoantibodies, resulting in a hypercoagulable state [1–3]. Clinically, APS presents through vascular thrombi in any tissue or organ or through pregnancy morbidity. APS is often secondary to other diseases or complications, such as systemic lupus erythematosus (SLE). Patients with APS may display a history of recurrent deep vein thrombosis, spontaneous abortion, and transient ischemic attacks. Laboratory classification criteria require one of the following antiphospholipid antibodies to exist over a 12-week period: lupus anticoagulant (LA), anticardiolipin antibodies (ACAs), or anti-beta-2-glycoprotein I (b2GP1) [4, 5].

Catastrophic antiphospholipid syndrome (CAPS), first described by Asherson in 1992, is an accelerated form of APS characterized by multiorgan failure that occurs in less than 1% of all APS cases [6–8]. Mortality rate was 37% in 2016, a decline of 13% from 50% in 2001 [9]. The current criteria for CAPS are the updated Sapporo criteria. This defines CAPS by the presentation of four preliminary criteria: (1) involvement of three or more organs, systems, and/or tissues, (2) development of manifestations simultaneously or in less than a week, (3) histopathological confirmation of small vessel occlusion, and (4) presence of antiphospholipid antibodies confirmed by two rounds of testing at least twelve weeks apart [10]. Several trigger factors for CAPS have been identified in literature. These include infections, major or minor surgery, neoplasms, lupus flare, obstetric complications, oral contraceptives, and withdrawal of anticoagulation [6, 8, 10, 11]. We describe a patient presenting with a new onset of CAPS after undergoing orthotopic liver transplant (OLT).

## 2. Case Report

We report the case of a 66-year-old woman who underwent an orthotopic liver transplant following decompensated liver cirrhosis complicated by hepatic encephalopathy and hepatocellular carcinoma (1.6 cm segment 8 single lesion: LR-5) (Model for End-Stage Liver Disease (MELD) score of 26). She had a medical history of alcohol-related cirrhosis, pancreatic body mucinous cystadenoma, colonic adenomatous polyps, and benign thyroid nodules. She had no history of hypercoagulable disease and no signs or symptoms of SLE at the time of transplant. However, preoperative lab workup a few months prior to transplant was positive for antinuclear antibodies. Once a suitable liver became available, the patient underwent an uncomplicated liver transplant utilizing a piggyback technique. The patient did not require packed red blood cell transfusions. Induction immunosuppression using standard methylprednisolone was started, while tacrolimus and mycophenolate were used for maintenance therapy. Steroid was tapered to 20 mg/day in the first week. The patient was discharged to a rehabilitation facility on postoperative day 9.

**Figure 1:**
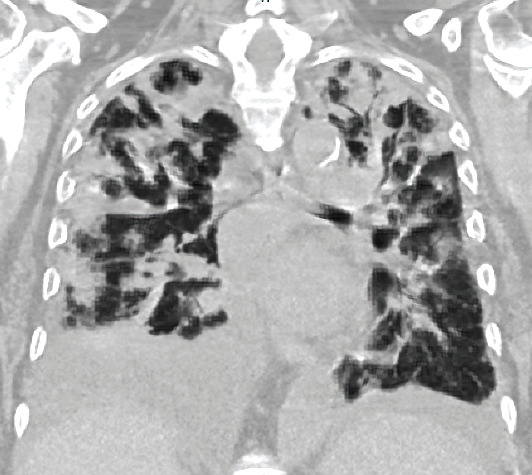
Chest CT scan at presentation to ED post-op concerning for ARDS.

Six days later, the patient was readmitted for worsening respiratory distress, fatigue, and increased oxygen requirements ongoing for the past three days. There was concern for possible pneumonia or pulmonary edema on arrival. With worsening respiratory insufficiency and bilateral infiltrates on CT imaging, the patient was intubated for acute respiratory failure (Figure 1). She was then started on broad-spectrum antibiotics and aggressive diuresis. Bronchoscopy with lavage and multiple COVID tests all resulted negative. She was diagnosed with acute respiratory distress syndrome (ARDS) and placed under intensive care. On admission, the patient had normal liver function. However, after admission, the patient had worsening transaminitis. Doppler ultrasonography on the third day following readmission revealed no arterial flow in the liver. Subsequent CT angiogram confirmed the lack of arterial flow and displayed an upper pole splenic infarct.  Table 1.

**Figure 2:**
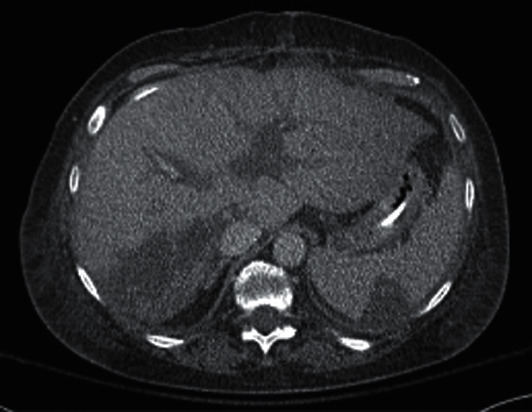
Splenic infarct visualized.

The patient was taken emergently to the interventional radiology suite for an angiogram with unsuccessful attempts at thrombolysis and revascularization. On the same day, the patient was then taken to the operating room, where the recipient's proper hepatic artery was found to be thrombosed and was subsequently resected with the transplant anastomosis. The resected tissue was sent for pathology, where the anastomosis appeared grossly patent. Chemical thrombolysis of the donor common hepatic artery with tissue plasminogen activator (tPA) was performed, resulting in backward flow of blood through the donor hepatic artery. The surgical team then constructed an infra-aortic jump graft using a cryopreserved donor iliac artery from the recipient aorta to the donor common hepatic artery. Intraoperative ultrasound then showed intrahepatic arterial flow in both left and right hepatic lobes and improvement in the hue of the liver. A tru-cut needle biopsy was then performed, showing mild to early perivenular hepatocyte dropout and hyperplastic hepatic arterioles. The patient was placed on therapeutic heparin infusion postoperatively. Liver function, lactate, and INR improved postoperatively, but a repeat ultrasound and CT angiogram two days post-op showed rethrombosis of the hepatic artery and occlusion of the jump graft, as well as new splenic infarcts (Figure 2). The patient's ARDS did not improve despite supportive measures, and a tracheostomy was performed.  Table 2.

A hypercoagulable workup displayed an increased anticardiolipin IgG titer of 113 CU (normal < 23). Further testing due to concern of APS displayed an increased beta-2-glycoprotein (IgG > 150, IgM 142) and positive LA. Given the findings, there was concern for generalized antiphospholipid syndrome or CAPS. A multidisciplinary meeting with rheumatology and intensive care was called resulting in a joint decision to start treatment with pulse steroids at 500 mg daily for three days. Plasmapheresis was also considered if the patient's condition did not improve but was avoided primarily due to hemodynamic instability and risk of infection. Improvement on steroid dose was noted. Patient was switched to therapeutic enoxaparin and bridged to warfarin. Thrombophilia workup showed 33% protein C activity, 49% antithrombin III activity, normal protein S activity, and no hypercoagulability mutations involving Factor V Leiden or prothrombin G20210. The patient also had multiple negative bacterial, viral, and fungal cultures. After completion of pulse steroids, the patient was placed on prednisone at 60 mg/day and was tapered to 20 mg/day over the next three weeks.

The patient continued to improve and was weaned off the ventilator thirty days after surgery. The patient was shortly after discharged to long-term acute care facility. Few weeks later, the patient was discharged home with trach decannulated. APS was confirmed on repeat lab testing 12 weeks after initial tests. The patient will be on life-long warfarin anticoagulation. She was recently seen in clinic on room air and ambulatory. INRs have been well controlled with continued outpatient warfarin management. The patient does not exhibit any signs of ischemic cholangiopathy and presents with normal liver function test.

## 3. Discussion

APS is an acquired multisystem autoimmune condition defined by vascular thromboses. It involves antiphospholipid antibodies (APL) directed at phospholipid-dependent clotting assays, negatively charged phospholipids, and their binding proteins resulting in an inhibition to their natural anticoagulant activity [12]. APS presents in a wide spectrum of primary, secondary, and catastrophic forms, with multiorgan failure in CAPS as the worst prognosis [4, 13]. CAPS is classified using the Sapporo criteria. The Sapporo criteria, the preliminary classification criteria for APS, were formulated in 1998 in Sapporo, Japan, and recommend the presence of one clinical and one laboratory criteria for the diagnosis of APS [14–16]. This includes either the presence of vascular thrombosis or pregnancy morbidity with the presence of anticardiolipin antibody or lupus anticoagulant or beta-2-glycoprotein on two or more occasions when tested at least 12 weeks apart [14]. These criteria were validated by Lockshin et al. in 2000 [15] and were updated by Miyakis et al. in 2006, recommending lab testing have a 12-week interval for the diagnosis of APS [16].

After the description of CAPS by Asherson in 1992, the preliminary criteria for its classification were formulated in 2002 [7] and recommend four criteria for the diagnosis of CAPS as well as to differentiate definite, where all 4 criteria are met, from probable CAPS. As previously mentioned, these include evidence of involvement of 3 or more organs, systems, or tissues; development simultaneously or within 1 week; histopathologic confirmation of small vessel occlusion in one or more organ; and laboratory confirmation of the presence of APL at least 12 weeks apart [7, 10].

Cases of CAPS following OLT have been reported previously, one posthumously, and demonstrate the importance of rapid recognition and escalation of care to prevent morbidities and mortality [6, 12]. All reported cases of CAPS concurrent with OLT have occurred in female patients aged 50-70. Our patient presented postoperatively with ARDS, hepatic artery thrombosis (HAT), and splenic infarction, all occurring within days. Additional histopathologic evidence of small vessel occlusion and the presence of APL was noted, thus meeting the criteria for a diagnosis of definite CAPS and prompting treatment with anticoagulation and steroids [3].

Collier et al. [2] present the first documented cases of APS in patients who received liver transplantation. The two presented patients suffered hepatic vascular thrombosis with the persistent presence of APL. In the first case, Collier et al. recorded the presence of abnormal levels of ACAs before the first transplant, after the second liver transplant, two months after the second transplant, and two months after the third transplant. The second patient was further investigated after the development of graft thrombosis, and high levels of both LA and raised ACAs were recorded after repeated testing. Although the APL panels after the thrombotic events were not completed 12 weeks apart and failed to demonstrate the updated criteria for persistence of APL, both of the presented patients had APL prior to their first liver transplantation. The first patient had elevated ACA levels a month prior to his first liver transplantation, and the second patient had elevated LA prior to her first liver transplantation. Notably, the patients' first transplantations failed due to graft thrombosis. This demonstrated that these patients suffered from definite APS through thrombosis and persistent APL [4]. The APS diagnosis indicates that the prior transplant failures may have been due to APS [2].

In conjunction to these case reports, Collier et al. completed a retrospective study of 132 orthotopic liver transplant recipients to determine if the presence of IgG anticardiolipin increases the risk of hepatic vessel thrombosis. No statistically significant association was established between the presence of ACAs alone and venous thrombosis, so thromboembolic prophylaxis in patients who only screened for IgG anticardiolipin was not recommended. However, patients who present with LAs and ACAs or patients with previous thrombotic events may benefit from prophylactic anticoagulation [2]. Bahaa El Din et al. [17] completed a small review of 40 patients who underwent liver transplantation and found while ACAs did not provide significant evidence of producing venous thrombosis, LAs were a statistically significant indicator of venous thrombosis. Furmańczyk-Zawiska et al. [18] completed a similar study determining whether the presence of APL would predict thrombotic risk and determined that the variable nature of ACAs make them an unreliable predictor of thrombotic events. Furmańczyk-Zawiska et al. concluded that monitoring patients with APL is not necessary in liver transplant patient.

Saunders et al. [13], agreeing with the multifactorial nature of thrombosis, promote a more aggressive approach in preventing further thrombotic events. Patients presenting with APL, especially that of LA, are under thrombotic risk and should be given prophylactic anticoagulation therapy. Saunders et al. emphasize the importance of utilizing bridging to maintain anticoagulation and incorporating both physical, intermittent pneumatic compression and/or gradual compression stock, as well as medical approaches to minimize thrombotic risks. The goal of therapy is to prevent the development of thrombotic events secondary to an inciting event, such as surgery or infection.

The CAPS Registry is a database of patients affected by CAPS created in 2000. It contains a disproportionate amount of women: 72% of recorded cases are women, with a mean age of 37 [19]. Interestingly, exactly half of the patients who presented with CAPS did not have a previous diagnosis of APS and nearly half (46%) developed CAPS without any history of previous thrombosis [9]. Most cases of CAPS (65.4%) were associated with an identifiable trigger event. These precipitating conditions included infections (46.7%), malignancy (17.6%), and, in 16.8% of these cases, surgical procedures [4, 20].

Villamil et al. [6] confirmed that multiorgan system failure, especially CNS compromise and renal involvement, is encountered in CAPS, and they recommend anticoagulation plus steroids as well as adjuncts of plasmapheresis and intravenous immunoglobulin for management. Asherson et al. [7] showed a survival benefit with initiating anticoagulation alone for treatment, but found better outcomes when used in combination with either steroids, plasma exchange, or intravenous gamma globulin, the so-called triple therapy. Infection was highlighted as the most prevalent precipitating factor for CAPS. Yasutomi et al. [1] report APS associated Budd-Chiari syndrome in an infant showing positive outcomes following living donor liver transplantation and the use of anticoagulation and immunosuppressive medication. This was shown to reduce measured b2GP1 IgG antibodies. Erkan et al. [11] looked at long-term outcomes of CAPS survivors from the CAPS Registry noting that on long-term high-intensity warfarin for anticoagulation there was no CAPS recurrence in this patient sample at 5.5 years after index CAPS event. In untreated patients, recurrence rate was 44-55%. 26% of the cohort developed further APS-related events with a mortality rate of 25% in this population. Long-term anticoagulation with warfarin was recommended.

Rodriguez-Pinto et al. [21, 22] studied a cohort of patients with CAPS to elucidate clinical and immunologic manifestations. From the study, the mortality rate was shown to be 37%. CAPS was shown to be precipitated by infection in younger patients and by malignancy in older patients when a precipitating factor was identified. In patients with SLE, severe cardiac and brain involvement was seen with a higher mortality of 48%. Otherwise, kidneys, lungs, brain, heart, and skin were organs involved in CAPS episodes. Antiphospholipid antibodies have been reported to be associated with certain entities involving the liver, e.g., primary biliary cirrhosis and autoimmune hepatitis A and B.

In our patient, pretransplant positive for ANA and antimitochondrial antibodies (AMA) could be related to a missed primary biliary cholangitis (PBC) which might be a risk factor for APS and CAPS. Steckelberg et al. [23] presented a patient with HAT following liver transplantation much like our own. This patient however had a previously documented thrombotic event with a diagnosis of APS. In their case study, the patient required retransplantation and had positive outcomes afterwards first on fondaparinux and then on long-term warfarin. Gologorsky et al. [12] reported a case of OLT associated with lethal intraoperative cardiac and aortic thromboses that was attributed to be secondary to CAPS on posthumous investigation.

Our patient did not require retransplantation; although despite anticoagulation after the second operation, she continued to suffer thrombotic complications of hepatic artery aortic jump graft and new splenic infarcts. Plasmapheresis was considered but avoided lest exchange affect the patient's hemodynamic instability. The patient also began to show improvement on just dual therapy of anticoagulation and steroids. Kazzaz et al. [9] discuss third-line options for refractory CAPS which include rituximab and eculizumab; however, there is less evidence supporting the efficacy of these therapies. Of note, we do not routinely use anticoagulation prophylaxis after liver transplant due to high risk of bleeding secondary to low platelets from hypersplenism and elevated INR. We use sequential compression devices and baby aspirin and early ambulation for DVT prophylaxis.

While our patient recovered from her acute illness, CAPS recurrence has been reported in 44-55% of patients who are not anticoagulated according to Erkan et al. [11]. Even with adequate treatment, patients with a history of CAPS were shown to have 40% risk of postoperative recurrence in that study. This data indicates a higher threshold for surgical intervention in patients with CAPS history. Surgeons should consider the high risk of recurrence when evaluating these patients preoperatively.

## 4. Conclusion

We report the case of a patient who suffered multisystem organ failure requiring prolonged intensive care stay due to CAPS following an orthotopic liver transplant. Although CAPS is a rare disease, it can lead to serious complications postliver transplant. We recommend APL testing if routine preoperative ANA and AMA tests are positive. A repeat APL test should then be performed in 12 weeks if positive to confirm the diagnosis of CAPS. If the Sapporo criteria are met, we recommend considering a treatment plan involving steroids and perioperative anticoagulation as first line.

## Figures and Tables

**Table 1:** Results of hematologic workup.

Lupus anticoagulant screen (STACLOT-La) (<8 secs)	9/1/21	11/23/21
Buffer	88	58.1
Delta	27.6	1
Phospholipid	60.4	57.1
Interpretation	Positive	Negative
		
Cardiolipin antibody	8/27/21	11/23/21
IgG (0-14)	113	<10
IgM (<12)		27
		
Beta-2-glycoprotein (<20)	9/1/21	11/23/21
IgA		89
IgG	150	116
IgM	142	47
		
	8/27/21	
Factor V Leiden	Negative	
Prothrombin G20210A panel	Negative	
		
Immunology	1/27/21	
ANA IgG	Detected	
Mitochondrial M2 antibody	Elevated	
SS-A/B antibody	Normal	
Smith antibody	Normal	

*Note*: the data for lupus anticoagulant screen is from patient chart review. The data for cardiolipin antibody is from patient chart review. The data for Factor V Leiden is from patient chart review. The data for prothrombin G20210A panel is from patient chart review. The data for immunology results is from patient chart review.

**Table 2:** Overview of reviewed literature.

Author	Background	Result	Conclusion
Aguiar and Erkan [4]	A stepwise approach for clinicians and researchers in the diagnosis of patients with CAPS	Development of algorithms for CAPS diagnosis in patients with and without history of APS or persistent APL positivity	It is critical to diagnose CAPS urgently when symptoms present, even without confirmatory APL tests
Asherson et al. [7]	Consensus criteria for definition and classification of CAPS	Formulation of the preliminary criteria for classifying CAPS	Guidelines to define CAPS and guide multimodal treatment therapy; anticoagulation with survival benefit in treatment
Cervera et al. [10]	Validation of the preliminary criteria for the classification of CAPS	Sensitivity, specificity, and positive and negative predictive values greater than 90% in classifying definite and probable CAPS	Use of the preliminary criteria for CAPS classification recommended
Collier et al. [2]	Retrospective study of liver transplant recipients with postoperative hepatic vessel thrombosis from APS	The comparison of anticardiolipin IgG presence in recipients with hepatic vessel thrombosis and recipients without did not show statistical significance	The presence of IgG anticardiolipin is not associated with increased risk of hepatic vessel thrombosis in liver transplant recipients with APS. Routine screening is not warranted
Erkan et al. [11]	Long-term follow-up for patients with CAPS after treatment	No recurrence of CAPS in patients on long-term high-intensity warfarin at 5.5 years; recurrence rate of 44-55% in untreated patients after first event	Long-term warfarin anticoagulation is recommended for long-term CAPS management
Gologorsky et al. [12]	Lethal multisystem organ failure following CAPS after liver transplantation	Clinical manifestation likely from previously undiagnosed APS complicated by use of antifibrinolytic therapy during liver transplantation	Caution with use of synthetic antifibrinolytics in liver transplantation especially in patients with viral hepatitis and APS
Kazzaz et al. [9]	Review of current approaches to diagnosis and treatment of CAPS	Anticoagulation and corticosteroids, IVIG, or plasma exchange recommended; cyclophosphamide use in patients with SLE	Triple therapy is marginally supported by retrospective data but recommended by most expert reviews
Lockshin et al. [15]	Validation of the Sapporo criteria for the classification of APS	Sensitivity, specificity, and positive and negative predictive values were 0.71, 0.98, 0.95, and 0.88, respectively	The Sapporo criteria for APS are usable for clinical studies
Miyakis et al. [16]	Update of the Sapporo criteria for the classification of APS	The laboratory criteria now require positive APL no less than 12 weeks apart vs. 6 weeks in previous criteria	Laboratory criteria was revised, use of “primary” and “secondary” APS was advised against, and CAPS was not discussed on here
Obed et al. [24]	Case of ACLF and BCS treated with LDLT from donor with APS	Improvement in multisystem organ failure after LDLT and anticoagulation	In cases of APS+ donors with no clinical manifestation, LDLT is safe and feasible
Reshetnyak et al. [5]	Liver transplantation in a patient with primary APS and BCS	Favorable outcomes following long-term use of dabigatran etexilate	Anticoagulation recommended for APS treatment
Rodriguez-Pinto et al. [21]	Review of the current management approach for CAPS	Recommended treatment for CAPS is anticoagulation, glucocorticoids, and plasma exchange or IVIG. Rituximab and eculizumab for severe and refractory CAPS	Triple therapy is the current best therapeutic approach
Rodriguez-Pinto et al. [22]	Clinical and immunologic manifestations of patients with CAPS	CAPS is majorly triggered by an event; kidneys most affected in multiorgan failure. Mortality rate is 37%	There are differences in CAPS patient presentation depending on age and presence of underlying chronic disease
Steckelberg et al. [23]	Complication of HAT after liver transplant in a patient with APS and BCS	Favorable outcomes following retransplantation and long-term anticoagulation	Prophylactic anticoagulation may be beneficial in liver transplant candidates with history of previous thrombotic event to prevent posttransplant HAT
Villamil et al. [6]	CAPS complicating orthotopic liver transplantation	CAPS causes multisystem organ failure. Anticoagulation plus steroid, plasmapheresis, and gamma globulin recommended	CAPS can be diagnosed when all other causes of multiorgan failure after transplant are ruled out
Wilson et al. [14]	Consensus criteria for definition and classification of APS	Formulation of the Sapporo criteria for classifying APS	Definite APS is considered present when at least 1 of the clinical criteria and at least 1 of the laboratory criteria are met
Yasutomi et al. [1]	APS-induced BCS in a 10-year-old child requiring liver transplant	Favorable outcomes following transplant and treatment with anticoagulation, steroids, and immunosuppression	Anticoagulation, steroids, and immunosuppression can be used in treatment of APS marked by a reduction in anticardiolipin antibody levels

CAPS: catastrophic antiphospholipid syndrome; APL: antiphospholipid antibodies; APS antiphospholipid syndrome; IgG: immunoglobulin G; IVIG: intravenous immunoglobulin; SLE: systemic lupus erythematosus; ACLF: acute on chronic liver failure; BCS: Budd-Chiari syndrome; LDLT: living donor liver transplant; HAT: hepatic artery thrombosis. Note: the data for Aguiar and Erkan is from Aguiar, C.L., and D. Erkan, *Catastrophic antiphospholipid syndrome: how to diagnose a rare but highly fatal disease*. Ther Adv Musculoskelet Dis, 2013. 5(6): p. 305-14. The data for Asherson et al. is from Asherson, R.A., et al., *Catastrophic antiphospholipid syndrome: international consensus statement on classification criteria and treatment guidelines.* Lupus, 2003. 12(7): p. 530-4. The data for Cervera et al. is from Cervera, R., et al., *Validation of the preliminary criteria for the classification of catastrophic antiphospholipid syndrome.* Ann Rheum Dis, 2005. 64(8): p. 1205-9. The data for Collier et al. is from Collier, J.D., et al., *Graft loss and the antiphospholipid syndrome following liver transplantation.* J Hepatol, 1998. 29(6): p. 999-1003. The data for Erkan et al. is from Erkan, D., et al., *Long term outcome of catastrophic antiphospholipid syndrome survivors.* Ann Rheum Dis, 2003. 62(6): p. 530-3. The data for Gologorsky et al. is from Gologorsky, E., et al., *Devastating intracardiac and aortic thrombosis: a case report of apparent catastrophic antiphospholipid syndrome during liver transplantation.* J Clin Anesth, 2011. 23(5): p. 398-402. The data for Kazzaz et al. is from Kazzaz, N.M., W.J. McCune, and J.S. Knight, *Treatment of catastrophic antiphospholipid syndrome.* Curr Opin Rheumatol, 2016. 28(3): p. 218-27. The data for Lockshin et al. is from Lockshin, M.D., L.R. Sammaritano, and S. Schwartzman, *Validation of the Sapporo criteria for antiphospholipid syndrome.* Arthritis Rheum, 2000. 43(2): p. 440-3. The data for Obed et al. is from Obed, A., A. Bashir, and A. Jarrad, *A case of live donor liver transplantation in acute-on-chronic liver failure with Budd-Chiari syndrome: donor and recipient with antiphospholipid antibody syndrome.* Am J Case Rep, 2018. 19: p. 767-772. The data for Reshetnyak et al. is from Reshetnyak, T.M., et al., *Liver transplantation in a patient with primary antiphospholipid syndrome and Budd-Chiari syndrome.* World J Hepatol, 2015. 7(19): p. 2229-36. The data for Rodriguez-Pinto et al. is from Rodriguez-Pinto, I., G. Espinosa, and R. Cervera, *Catastrophic antiphospholipid syndrome: the current management approach.* Best Pract Res Clin Rheumatol, 2016. 30(2): p. 239-249. The data for Rodriguez-Pinto et al. is from Rodriguez-Pinto, I., et al., *Catastrophic antiphospholipid syndrome (CAPS): descriptive analysis of 500 patients from the International CAPS Registry.* Autoimmun Rev, 2016. 15(12): p. 1120-1124. The data for Steckelberg et al. is from Steckelberg, R.C., Z.D. Antongiorgi, and R.H. Steadman, *Liver transplantation in a patient with antiphospholipid syndrome: a case report.* A A Case Rep, 2017. 9(5): p. 148-150. The data for Villamil et al. is from Villamil, A., et al., *Catastrophic antiphospholipid syndrome complicating orthotopic liver transplantation.* Lupus, 2003. 12(2): p. 140-3. The data for Wilson et al. is from Wilson, W.A., et al., *International consensus statement on preliminary classification criteria for definite antiphospholipid syndrome: report of an international workshop.* Arthritis Rheum, 1999. 42(7): p. 1309-11. The data for Yasutomi et al. is from Yasutomi, M., et al., *Living donor liver transplantation for Budd-Chiari syndrome with inferior vena cava obstruction and assoiciated antiphospholipid antibody syndrome.* J Pediatr Surg, 2001. 36(4): p. 659-62.

## Data Availability

The secondary data used to support the findings of this study are included within the article, in References.
